# Institutional Constraints as an Obstacle for Prioritizing Nursing Interventions During the COVID-19 Pandemic—Critical Care Nurses’ Experiences

**DOI:** 10.1177/23779608221133656

**Published:** 2022-10-31

**Authors:** Åsa Engström, Angelica Fredholm, Anna Nordin, Maria Andersson

**Affiliations:** 1 Department of Health, Education and Technology, Division of Nursing and Medical Technology, Lulea University of Technology, Luleå, Sweden; 2 County Council, Värmland, Sweden; 3 Department of Health Science, Faculty of Health, Science, and Technology, Karlstad University, Karlstad, Sweden; 4 Swedish Red Cross University College, Huddinge, Sweden

**Keywords:** COVID-19, critical care, nursing intervention

## Abstract

**Introduction:**

The demands of the pandemic such as staff shortages and limited resources combined with new guidelines regarding infection control may have required the prioritizing of nursing interventions.

**Objectives:**

The aim of this study was to describe critical care nurses’ experiences of prioritizing nursing interventions for patients with COVID-19 in intensive care units (ICUs) during the pandemic.

**Method:**

A qualitative descriptive study was gathered from open-ended questions included in a cross-sectional online questionnaire. Characteristics were presented using descriptive statistics, and open-ended questions were analyzed using qualitative content analysis with an inductive approach. The study was conducted in Sweden and focused on critical care nurses working in ICUs during spring 2021 and the second year of the COVID-19 pandemic.

**Results:**

During the COVID-19 pandemic, 87% of the critical care nurses had provided orientations for new co-workers, and 52% had supervised intensive care nursing students. In all, 70 answered the question of whether they had prioritized nursing care differently during the pandemic; 86% reported that they had and 14% had not. The qualitative analysis resulted in one theme, Institutional constraints as an obstacle for nursing interventions, with three categories: Prioritizing lifesaving interventions, Performing nursing interventions less frequently, and Not able to provide the nursing care I wish to provide.

**Conclusion:**

Institutional constraints as an obstacle for nursing interventions is the overall theme. It illustrates how critical care nurses have been forced to prioritize, thereby not being able to provide the nursing interventions they wanted to do provide, and it describes their feelings in this situation. The nurses need recovery and possibilities for reflection. The organization must also recover and not only return to how it was before the pandemic but also to learn from recent events and take actions to reduce the long-term effects on staffing.

## Introduction

During the COVID-19 pandemic, critical care nurses (CCNs) in intensive care units (ICUs) have had to decide what and how much nursing care they could provide to patients. Worldwide, national guidelines were developed for how to prioritize intensive care treatments for critically ill patients with COVID-19 diseases (Halpern et al., 2020; Leclerc et al., 2020). However, these guidelines were mostly medical and, according to Bergman et al. (2021) and Sezgin et al. (2021), there were no guidelines for how to prioritize nursing interventions for patients with COVID-19 diseases. Therefore, prioritizing nursing interventions had to be done by the CCNs themselves.

The COVID-19 pandemic has placed enormous pressure on ICUs (Selman et al., 2020), and CCNs have had to prioritize between different nursing interventions (Bergman et al., 2021; Sezgin et al., 2021). They describe nursing care as suboptimal (Andersson et al., 2022a; Bergman et al., 2021; Boulton et al., 2021; Cadge et al., 2021; Maaskant et al., 2021) and challenges including working with new colleagues and maintaining existing work relationships, organizational-level obstacles such as lack of staff and limited resources (Andersson et al., 2022a; Bergman et al., 2021; Cadge et al., 2021; Vincent & Creteur, 2020) and visiting restrictions for relatives (Andersson et al., 2022a; Bergman et al., 2021; Boulton et al., 2021; Maaskant et al., 2021). As a result of the latter, CCNs had less contact with relatives, and they described how patients treated in ICUs felt less like persons (Andersson et al., 2022a; Bergman et al., 2021; Boulton et al., 2021; Maaskant et al., 2021).

## Review of the Literature

Nursing care is based on a humanistic view of each patient as unique and as an active and creative part of the environment (Ricoeur, 1994; Swedish Society of Nursing, 2011; WHO, 2016). By acknowledging the person as a whole and attempting to meet the patient's biological, psychological, social, relational, and spiritual needs (Halvorsen et al., 2009), nursing care is provided in a holistic manner. However, a systematic review (Mandal et al., 2020) found that nursing interventions, including meeting patients’ emotional, educational, mobility, and hygiene needs, face a lower priority related to other nursing interventions when resources are limited and due to the organizational environment. Requirements in regard to nursing interventions influenced the registered nurses’ job satisfaction, increased their intention to leave their profession and high turnover, and affected patient outcomes (Mandal et al., 2020).

The ethical nature of nursing care is associated with wishing others well, which is a form of give and take that relies on the recognition of the other as a unique person (Ricoeur, 1994). Person-centered care (PCC) has been described as bringing the person back into care (Edvardsson, 2015; Ekman et al., 2011) and has become a key of quality care in many developed countries (Hanefeld et al., 2017). The care environment has the potential to support or to restrict PCC (Moore et al., 2017) and, according to Crilly et al. (2019), CCNs have sometimes experienced that the technology in ICUs appears to be more important than the patients. During the first phase of the COVID-19 pandemic, CCNs reported being ambitious and knowledgeable about PCC, but obstacles prevented them from providing it (Andersson et al., 2022a). Having to compromise PCC (Andersson et al., 2022a), patient safety and quality of care (Bergman et al., 2021; Cadge et al., 2021; De Boer et al., 2015) might trigger moral distress for CCNs, which arises when one knows the right thing to do, but institutional constraints make it nearly impossible to pursue the appropriate course of action (Jameton, 1993). Professional identity can be viewed as a lived experience where this identity is a product of constant change and negotiation of meaning. The negotiation of meaning is about what matters and which values shape our belonging to a professional community (Wenger, 1998). Thus, having to compromise quality of care can negatively affect nurses. Moradi et al. (2021) describe CCNs’ challenges with provision of care for patients with COVID-19 as organization's inefficacy in supporting nurses, physical exhaustion, living with uncertainty, and psychological burden of the disease. Italy was one country that was affected severest by the COVID-19 pandemic and the healthcare staff were highly burdened (Lasalvia et al., 2021).

In summary, due to the demands of the pandemic, which included shortages of CCNs and limited time and resources combined with new guidelines regarding infection control, it may have been necessary to prioritize nursing interventions. According to Mandal and Seethalakshmi (2019), prioritizing in nursing care is seen as rationing of care or missed care. It implies aspects of required patient care that is delayed or omitted. The phenomenon is closely related to patient safety and quality of nursing care (Mandal & Seethalakshm, 2019).

However, little is known about how CCNs experienced having to prioritize between different nursing interventions in the ICU during the COVID-19 pandemic. This study asks CCNs how they experienced prioritizing nursing interventions in the face of new challenges, which included obstacles such as limited resources and work organization. Therefore, the aim of this study was to describe CCNs’ experiences of prioritizing nursing interventions for patients with COVID-19 in ICUs during the pandemic.

## Methods

### Design

A qualitative descriptive study was gathered from open-ended questions included in a cross-sectional online questionnaire. The study followed the checklist for reporting results of internet surveys (CHERRIES) (Eysenbach, 2004). The method was chosen as a suitable method during the COVID-19 pandemic because of recommended distancing to minimize the physical contact between people and thereby reducing the risk of the spread of the highly contagious nature of the COVID-19 illness (European Centre for Disease Prevention and Control, 2020). The study is derived from a major project.

### Research Question

How do CCNs experience prioritizing nursing interventions in the face of new challenges as changed work organizations and limited resources?

### Sample

The study was conducted in Sweden and included CCNs who were working in ICUs during the second year of the COVID-19 pandemic. In Swedish ICUs, the nurse-to-patient ratio is normally 1:1-2 and the ICU team caring for critically ill patients consists of CCNs, enrolled nurses, specialist physicians, and physiotherapists. However, during the COVID-19 pandemic, the competence mix in the ICU-team had to be changed and included registered nurses without post-graduate education in intensive care.

### Ethical Approval

The participants received information concerning the study's aim, confirmation that participation was voluntary and that their identity would be kept confidential. Informed consent was presumed by answering the questionnaire and participants agreed to the terms of publishing the results. This procedure corresponds to the World Medical Association’s (2020) ethical principles. Ethical approval was not needed since the Swedish Ethical Review Act includes only studies that handle sensitive data and patient data.

CCNs employed in Swedish ICUs as registered nurses and who had a post-graduate education in intensive care at an advanced level (Marshall et al., 2017) were included in the study. In Sweden, this post-graduate education includes a one-year academic program, *Study Program for Specialist Nursing in Intensive Care* (60 ECTS credits) with a master's degree.

### Data Collection

The questionnaire package comprised of ten questions and one instrument: the Italian version of MDS-R (Lamiani et al., 2017). The results of MDS-R are reported in the study by Andersson et al. (2022b). Eight questions asked about demographic characteristics such as sex, age, years of work experience in an ICU, and education, as well as job-related questions about increased responsibility, provided orientation for new co-workers, supervision of intensive care nursing students, and prioritization of nursing interventions. The qualitative data were collected through the possibility of describing in free text and with no word limit their experiences in relation to the open-ended questions “*Can you give examples of how you have prioritized nursing interventions?”* and “*Can you explain the reason for your prioritization?”*

Data collection took place between May and June of 2021, and participants were recruited through an announcement posted on nursing groups on the Facebook social media platform. Information about the study and a link to the questionnaire were presented on the Facebook pages of the Swedish Association for Anaesthesia and Critical Care Nurses, The Intensive Care Nurse, and Registered Nurse. Two reminders were posted on each web page. Each participant could complete the questionnaire only once.

### Data Analysis

Twenty-seven (38%) of the participating CCNs were involved in both the quantitative and qualitative parts. Quantitative characteristics were examined using descriptive statistics. Qualitative data from the open-ended questions were analyzed using qualitative content analysis with an inductive approach. The answers to the questions were considered as one written text. The content analysis was performed in a number of steps in which the manifest content of the text was preserved (Graneheim & Lundman, 2004). The text was read several times to gain a sense of the content as a whole. The entire text was then reread to identify meaning units, guided by the aim of the study. The meaning units were condensed and sorted into categories related by content, thus constituting an expression of the manifest content of the text. By moving back and forth between the text and the results of the analysis, we progressively refined the findings. The three final categories were then compared, and a theme, that is, threads of meaning that appeared in the categories, was identified (Graneheim & Lundman, 2004). Creating a theme is a way to connect the underlying content within the categories, and a theme can be defined as the expression of the latent content of the text, while a category can be described as the expression of the manifest content (Graneheim & Lundman, 2004). We analyzed the transcriptions independently and compared our findings before reaching a final agreement. Lastly, the transcripts were read once more to confirm and validate the theme and the categories.

## Results

### Sample Characteristics

A total of 71 CCNs answered the questionnaire, and 82% were women. Most of the participants (73%) were  ≥ 36 years of age; 97% had a post-graduate education in intensive care; and 53% had  ≤ 10 years’ experience working in ICUs (see Table 1).

**Table 1.** Characteristics of CCNs (*n*  *=*  71) Working in ICUs.

Variable	*n* (%)
Sex	
Female	58 (82)
Male	11 (16)
Unknown	2 (3)
Age in years	
≤25	1 (1)
26–35	18 (25)
36–45	25 (35)
46–55	19 (27)
≥56	8 (11)
Education	
Intensive care (60 ECTS)	69 (97)
Not in intensive care	2 (3)
Years of experience in ICU	
≤5	22 (31)
6–10	16 (22)
11–15	14 (20)
≥16	19 (27)

CCN = critical care nurse; ICU = intensive care unit.

A majority of CCNs (86%) experienced having prioritized nursing care differently during the COVID-19 pandemic. Almost all of them (97%) also experienced increased responsibility for nursing care; 87% had oriented new co-workers to the ICU and its regimen, and 52% also supervised intensive care nursing students (see Table 2).

**Table 2.** CCNs’ (*n*  *=*  71) Experiences of Prioritizing Nursing Interventions, Increased Responsibility, Orientation of New Co-workers and Supervision of ICU Nursing Students During the COVID-19 Pandemic.

Questions	*n* (%)
Have you made nursing intervention priorities differently during the COVID-19 pandemic?	
Yes	60 (86)
No	10 (14)
Do you consider yourself as having taken on greater responsibility during the COVID-19 pandemic?	
Yes	69 (97)
No	2 (3)
Have you provided orientation to new co-workers during the COVID-19 pandemic?	
Yes	62 (87)
No	9 (13)
Have you supervised intensive care nursing students during the COVID-19 pandemic?	
Yes	37 (52)
No	34 (48)

CCN = critical care nurse; ICU = intensive care unit.

The CCNs stated that the prioritization of nursing interventions was at its worst during spring 2020 and the first phase of the COVID-19 pandemic. They felt hopeful about the future and, as time passed, they felt that they could provide nursing interventions in a more effective way. In addition, they described how their competence as CCNs increased as a result of the greater responsibilities they had to assume. The written text has been analyzed and the findings are presented as one theme, “Institutional constraints as an obstacle for nursing interventions,” with three categories: *Prioritizing lifesaving interventions, Performing nursing interventions less frequently,* and *Not able to provide the nursing care I wish to provide*.

#### Institutional Constraints as an Obstacle for Nursing Interventions

The theme “Institutional constraints as an obstacle for nursing interventions” emerged from the three categories. The theme runs like a thread through the categories, and findings describe how lack of time and resources were common reasons for not performing the nursing interventions in a timely manner or, sometimes, not doing them at all. The CCNs described not having adequate time to make assessments and carry out the measures they would in ordinary cases. There was also perceived insecurity regarding the competence of new co-workers from other fields of nursing. With the need to supervise these co-workers and students, there were fewer opportunities to help patients become mobile due to lack of time, lack of staff, or a feeling of insecurity during the work shift. “*Much that you wanted to do, you did not have time to do*” was a common description, and the CCNs felt frustrated when they could not care for patients as they wished to because they had to prioritize lifesaving interventions. The CCNs also missed opportunities to meet and talk with patients’ relatives, which is considered a prerequisite for PCC. Without this person-centeredness, nursing was perceived as mechanic. The personal-protective equipment was also a source of creating a distance to patient and relatives. Having to delegate nursing interventions to less qualified co-workers was a source of frustration. They felt exhausted by what they had gone through and mentioned a feeling of knowing what they wanted and could provide for patients and their relatives, but not having possibilities to do so. The CCNs also stated that the situation was at its worst during the first phase of the COVID-19 pandemic and that it then became a bit better.

### Prioritizing Lifesaving Interventions

CCNs prioritized lifesaving interventions above non-direct life-sustaining interventions. Nursing interventions such as pressure relief, mobilization, wound dressings, oral care, and calculating patients’ nutritional needs were left undone during certain work shifts due to high workloads and the need to prioritize. Other examples of nursing interventions that were not performed included changing hoses to respirators, setting arterial pressure, and documentations. CCNs also noted that less severely ill patients received less nursing care and how nursing interventions were commonly deprioritized in favor of medical interventions:

Changes of body positions have been deprioritized, it was longer intervals due to unstable circulation and respiration.

When I have been responsible for many patients, it has happened that I have thought about who has had the greatest chance of surviving in order to be able to prioritize—awful!

I have been responsible for more patients; then, it is not possible to maintain a high level of nursing. The medical priority has to come first.

There was no time to perform all the nursing measures I wanted to perform—washing patients’ hair, turning them, mobilization. The list goes on.

Nutrition is another thing—not having time to calculate what speed gruel or total parenteral nutrition needs to go in to meet the patient’s energy needs.

Infrequent replacement of filters for respirators or no replacement of suction systems when lacking time.

### Doing the Nursing Interventions Less Frequently

CCNs described providing nursing interventions less frequently compared to what they used to do or what was ordered or suggested, and interventions had to be adapted to the care environment. In addition, they did not have enough time to change patients’ positions in beds; moreover, patients with COVID-19 required more careful monitoring because a slight side turn of position sometimes led to deterioration in a patient's oxygen saturation. Therefore, the nursing intervention to change patients’ positions was performed only when CCNs were confident that the ICU staff numbers were adequate for checking on oxygen saturation levels. Likewise, the retention checks for feeding tubes took place later than every 6 h.

Abdominal position meant that other nursing measures had to wait

Infrequent turning, adjustment and patients’ changes of position, usually unknowingly, were not discovered until later due to the lack of time.

Everything that we usually do, from shaving and combing patients’ hair to changing a dirty shirt, was not done often enough. But in the worst cases, I have also felt that common position changes, oral care, pressure ulcer prophylaxis, replacement of tubes and more were de-prioritized or postponed, and then absent, and also that abdominal-position patients were not turned onto their backs in time.

Not able to do the nursing care I wish to do.

CCNs described how they were not able to perform the kind of nursing care they wanted to, and there was no time to do anything extra for the patients. They depicted providing nursing care as being on an assembly line, where the patient as a person disappeared. There were examples of nursing interventions they were unable to do, for example, writing in diaries for patients.

Several of the CCNs reported difficulties contacting patients’ relatives due to visiting restrictions and contact by phone as being hindered by personal protective equipment. They had to remain at patients’ bedsides, and often it was not possible to change their clothes and face masks and leave the patient's bedside to talk to relatives by phone outside the ICU. Consequently, CCNs did not have opportunities to get to know their patients as individuals with help from their relatives. Furthermore, the CCNs described how the lack of adequate staff resulted in their being responsible for as many as three times the number of patients they ordinarily cared for, and therefore, they could not provide the nursing care they wanted to. They also felt forced to delegate nursing interventions to co-workers from other units and specialties, who provided nursing interventions without sufficient knowledge and competence, leading the CCNs to be concerned about reduced patient safety and quality of care.

You wished you had time to provide better care. The time is not enough to do the little extra for the patients. You barely have time to do what you have to do.

Lack of time, there is no time to do the little extras; without them, it is just basic nursing care and sometimes there is not even time for that.

Since then, conversations with relatives have often been prioritized away, and responsibility for this has been left to doctors, which has meant that you do not get the same understanding about who you are caring for.

Borrowed staff will perform many of my regular tasks, and then there is a lack of quality. They do not have the competence to continuously make assessments in the work with seriously ill patients simply because they do not have specialist competence.

## Discussion

The aim of this study was to describe Swedish CCNs’ experiences of prioritizing nursing interventions for patients with COVID-19 in ICUs during the pandemic. A majority of CCNs (86%) prioritized differently, and nursing interventions were de-prioritized in favor of medical interventions because of institutional constraints. The essence of nursing care within the PCC framework by McCormack and McCance (2016) reflects humanistic caring with moral components such as mutual respect and understanding, and not being able to provide PCC nursing care has the potential to cause moral distress (Jameton, 1993). Moral distress is well-known to affect moral integrity and the ability to deliver care with quality (Oh & Gastmans, 2015), and this problem was commonly described.

The COVID-19 pandemic has added additional stressors (Azoulay et al., 2020) to the already complex and demanding nursing care required in ICUs and, according to Sacco et al. (2015), these challenging circumstances are causal factors of compassion fatigue. Compassion fatigue, first identified in nursing care by Joinson (1992), describes nurses’ responses of either emotional distancing to control their emotions or feeling helpless and angry as they watch patients suffer through severe illnesses. Such situations might negatively affect nurses’ well-being and reduce the quality of the nursing care provided.

Previously, Winch et al. (2015) confirmed that the ability to practice with compassion is a complex intercourse between nurses, for example CCNs, and the environment in which they work. Nurses might base their decisions about whether to postpone primary nursing interventions on their perceived ability to control situations in their work environment (Abdelhadi et al., 2022). Factors that may reduce compassion fatigue are agreements about nursing interventions that are central for showing compassion, such as nursing care plans consistent with CCNs’ understanding of quality care, and connections with patients and relatives (Jones et al., 2020). The CCNs in the present study described nursing care as being performed on an assembly line and having limited contact with patients’ relatives. Our findings indicate that there seems to be an increased risk for moral distress and compassion fatigue among the CCNs because of the work environment, over which they had little control. Further research is needed in this area for a deeper understanding of this phenomenon.

No CCN in the present study mentioned the existence of any guidelines to help nurses prioritize their nursing interventions, and this is in line with previous studies (Bergman et al., 2021; Sezgin et al., 2021). According to the American Nurses Association (ANA, 2020), CCNs’ decisions must be supported by the systems in which they provide nursing care.

CCNs had to prioritize which nursing interventions they could safely delegate to co-workers who lacked intensive care experience. At the same time, CCNs were orienting, supervising, and leading a team of nurses without specific intensive care competence, while they were also in charge of patient care (Andersson et al., 2022a; Cadge et al., 2021; Montgomery et al., 2021). This situation generated additional cognitive and emotional demands (Montgomery et al., 2021). According to Montgomery et al. (2021), non-intensive care nurses also faced challenges in adapting to unfamiliar language, terms, and processes, and some were not well-equipped to perform even basic tasks such as recording observations or washing patients. Further investigation of how new co-workers without intensive care experiences describe their experiences working in ICUs during the COVID-19 pandemic is needed.

Moreover, the shortage of CCNs meant that those who were providing nursing care in ICUs were required to take on even greater responsibilities, and concurrently, they needed to trust others who came into the ICU to work. Nurse anesthetists who worked in ICUs during the COVID-19 pandemic have described feeling ambivalent about their work in the ICU. They lacked information, which led to feelings of uncertainty and resulted in expectations that did not correspond to the reality. They reported that, because of an inadequate introduction to the ICU protocols, they could only provide “sufficient” care, which in many cases caused ethical stress. Not being able to get to know their new colleagues well enough to build effective cooperation led to frustration (Hallgren et al., 2022). Therefore, the COVID-19 pandemic can be described as an extreme period in health care, and this may be especially true for those who have worked in intensive care either with or without previous experience or education.

### Prioritizing Lifesaving Interventions

CCNs prioritized life-saving interventions rather than basic nursing interventions such as mobilization, oral care, and calculating patients’ nutritional needs. The primary goal for intensive care is to prevent further physiological deterioration, supporting vital organs, and sustaining life during a period of life-threatening organ-system insufficiency (Marshall et al., 2017). In life-and-death situations, CCNs’ reduced prioritization of basic nursing care might, in fact, be required in order to deliver safe, effective nursing care. However, the long-term consequences of not providing nursing care might also result in life-threatening situations and suffering because when essential aspects of nursing care are delayed, incomplete, or lacking entirely, there are potentially negative implications for numerous patient outcomes, and patient safety is at risk (Kalisch, et al., 2009).

CCNs described a rapid acceleration in levels of responsibility. Larsson et al. (2022) highlighted the need for daily reflection on patient safety, workload, and work environment in the ICU, something that seemed to be lacking during the pandemic, according to the CCNs in our study. During the pandemic, the primary role of CCNs changed to one where many more patients would be monitored and where staff, sometimes with unclear skills, would be monitored and supervised. This reduced the possibility for PCC, both organizationally and from a nursing perspective. It can also be seen as risky due to the established link between low nurse competence and staffing and increased mortality for patients (Aiken et al., 2014).

The CCNs reported that they performed the nursing interventions less often or not at all compared to what they used to do before the pandemic. Mills and Duddle (2021) studied missed nursing care interventions in Australia. They found that the main contributing factors to missed nursing care were inadequate staffing, environmental factors, and urgent situations, which is rather similar to our findings.

Kalánková et al. (2020) found that missed, rationed, and unfinished nursing care negatively influenced patient outcomes in the context of patient safety and quality of nursing care. It has been shown that increased nurse staffing is associated with a decrease in missed care; in other words, adequate nurse staffing leads to reduced unmet nursing needs and improved patient outcomes (Jones et al., 2020). Arman and Rehnsfeldt (2007) found that being able to do the “little extras” within nursing care has the power to preserve dignity and make patients feel they are valued. It also offers hope by witnessing goodness that has the power to open up both patients and nurses to develop and grow in their understanding of life. However, in our study, the CCNs faced obstacles even in the provision of basic nursing care, which probably negatively affected the patients as well as the CCNs.

## Strengths and Limitations

In this study, participants comprised a non-probability sample from three different nursing groups on a social media platform. The nursing groups on Facebook's social media platform were public and, therefore, might have followers from other specialties as well. Those who participated wanted their voices to be heard. Due to the rapid pace of social media and the vast amount of information, it is unlikely that all followers of the nursing groups had the possibility to pay attention to the request. Also, the announcement about the study and link to the questionnaire was made on professional-specific sites.

The data in the present study was gathered from closed- and open-ended questions in a cross-sectional online questionnaire. To understand the phenomenon of prioritizing nursing interventions in depth, interviews face-to-face with CCNs are needed. However, the richness of the data from the open-ended questions speaks of an urgent need from participants to share their experiences, which contributes to the credibility of the study. Also, the nature of answers to the open-ended questions presupposes a first-hand experience of the phenomena under study, thus increase rigor.

## Conclusion

The COVID-19 pandemic added additional stressors to the already complex and demanding work of providing nursing care in ICUs. Institutional constraints as an obstacle to nursing interventions are this study's overall theme illustrating how the CCNs felt that they were forced to prioritize and not provide nursing interventions they saw a need for and wanted to provide but could not. It also describes the feelings this elicited for the CCNs. What they describe are situations and experiences that represent high levels of moral distress, and the risk for compassion fatigue is obvious. In the war against COVID-19, CCNs have been in the frontlines, lacking colleagues and equipment. They now need possibilities to recover, but also prerequisites to be able to perform the nursing interventions that are needed.

## Implications for Practice

Clinical implications are possible to discern at both an individual and organizational level. The CCNs have a need for recovery with possibilities for reflection and support. The organization must also recover and not only return to how it was before the pandemic but also to learn from recent events and take actions to reduce the long-term effects on staffing, work-related and stress-induced illness, etc. On a national level, there is a need to address the state of intensive care as a whole. There is also a need to follow up, and study, how missing nursing interventions during the pandemic influenced patients and their relatives.
